# Potency- and Selectivity-Enhancing Mutations of Conotoxins for Nicotinic Acetylcholine Receptors Can Be Predicted Using Accurate Free-Energy Calculations

**DOI:** 10.3390/md19070367

**Published:** 2021-06-25

**Authors:** Dana Katz, Michael A. DiMattia, Dan Sindhikara, Hubert Li, Nikita Abraham, Abba E. Leffler

**Affiliations:** 1 Schrödinger, Inc., 120 West 45th St., New York, NY 10036, USA; dana.katz@schrodinger.com (D.K.); michael.dimattia@schrodinger.com (M.A.D.); dan.sindhikara@merck.com (D.S.); hubert.li@schrodinger.com (H.L.); 2 D.E. Shaw India Private Ltd., Hyderabad 500096, India; nikita.abraham@schrodinger.com

**Keywords:** conotoxin, nicotinic acetylcholine receptor, selectivity, free-energy perturbation

## Abstract

Nicotinic acetylcholine receptor (nAChR) subtypes are key drug targets, but it is challenging to pharmacologically differentiate between them because of their highly similar sequence identities. Furthermore, α-conotoxins (α-CTXs) are naturally selective and competitive antagonists for nAChRs and hold great potential for treating nAChR disorders. Identifying selectivity-enhancing mutations is the chief aim of most α-CTX mutagenesis studies, although doing so with traditional docking methods is difficult due to the lack of α-CTX/nAChR crystal structures. Here, we use homology modeling to predict the structures of α-CTXs bound to two nearly identical nAChR subtypes, α3β2 and α3β4, and use free-energy perturbation (FEP) to re-predict the relative potency and selectivity of α-CTX mutants at these subtypes. First, we use three available crystal structures of the nAChR homologue, acetylcholine-binding protein (AChBP), and re-predict the relative affinities of twenty point mutations made to the α-CTXs LvIA, LsIA, and GIC, with an overall root mean square error (RMSE) of 1.08 ± 0.15 kcal/mol and an R^2^ of 0.62, equivalent to experimental uncertainty. We then use AChBP as a template for α3β2 and α3β4 nAChR homology models bound to the α-CTX LvIA and re-predict the potencies of eleven point mutations at both subtypes, with an overall RMSE of 0.85 ± 0.08 kcal/mol and an R^2^ of 0.49. This is significantly better than the widely used molecular mechanics—generalized born/surface area (MM-GB/SA) method, which gives an RMSE of 1.96 ± 0.24 kcal/mol and an R^2^ of 0.06 on the same test set. Next, we demonstrate that FEP accurately classifies α3β2 nAChR selective LvIA mutants while MM-GB/SA does not. Finally, we use FEP to perform an exhaustive amino acid mutational scan of LvIA and predict fifty-two mutations of LvIA to have greater than 100X selectivity for the α3β2 nAChR. Our results demonstrate the FEP is well-suited to accurately predict potency- and selectivity-enhancing mutations of α-CTXs for nAChRs and to identify alternative strategies for developing selective α-CTXs.

## 1. Introduction

Nicotinic acetylcholine receptors (nAChRs), members of the pentameric ligand-gated ion channel family and commonly referred to as Cys-loop receptors [1], are divided into muscle-type and neuronal-type. Neuronal-type receptors are homopentamers or heteropentamers of various subunit compositions. Each interface is made up of the principal side (+) of an α subunit (α2–α10) and the complementary side (−) of an α subunit (α7, α9) or β subunits (β2–β4) in homo and heteropentamers, respectively, which have distinct biophysical and pharmacological properties [2,3]. Subunits are composed of an extracellular domain (ECD), a transmembrane domain (TMD), and an intracellular domain arranged similarly to barrel staves to form a cation-conducting central pore. The binding pocket for acetylcholine lies in the “orthosteric site” at the interface of adjacent principal and complementary subunits in the extracellular domain. The nAChRs play important roles in vital physiological processes (α3β2 nAChR) and in diseases, including schizophrenia (α7 nAChR), addiction (α3β4 nAChR, α4β2 nAChR), and pain (α9α10 nAChR) [4,5,6]. The high degree of sequence identity between interchangeable nAChRs subunits (57–70%) makes selective inhibition of a specific nAChR difficult and increases the risk of unwanted side effects due to activity at off-target nAChRs [1].

α-Conotoxins (α-CTXs) are small, disulfide-rich peptides (usually ~12 to 20 amino acids in length) that are isolated from the venom of predatory marine cone snails, which have attracted special interest as possible nAChR therapeutics and tool compounds due to their ability to discriminate between similar nAChRs [7,8,9]. These conotoxins bind to orthosteric sites on nAChRs and function as competitive antagonists by inhibiting activity of the channel [7]. The so-called “4/7” α-CTXs have been proven to be particularly adept at discerning differences between closely related nAChRs [10]. NMR structures have revealed that these conotoxins contain two disulfide bonds with four residues in the first intercysteine loop (loop 1) and seven residues in the second intercysteine loop (loop 2), as well as a variable number of N-terminal residues and often C-terminal post-translational modifications. Some principles for improving the selectivity of these conotoxins via mutagenesis have begun to emerge [11], such as focusing efforts on loop 2 vs. loop 1. In addition, the locations and thermodynamics of water sites in the peptide toxin binding pockets of ion channels, as computed by inhomogeneous solvation theory implemented in the WaterMap algorithm, have recently been shown to explain structure–activity relationships (SAR) for bungarotoxin and the muscle-subtype nAChR [12]; however, accurately predicting selectivity-enhancing mutations is still an arduous process with much uncertainty that would benefit from new approaches [13].

**Figure 1:**
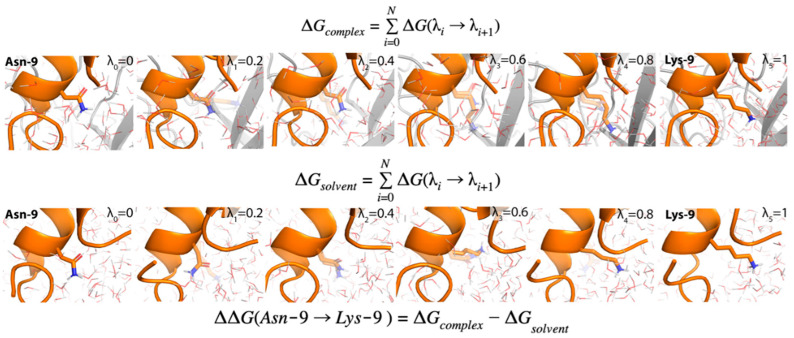
FEP calculation of the relative binding free energy due to a mutation. The peptide being mutated is represented in orange and the receptor to which it is bound is depicted in gray. Water molecules are shown as lines, with oxygens colored red and hydrogens colored white. The λ window is shown in the upper right-hand corner of each frame. In particular, λ = 0 represents the unmutated sidechain (Asn-9, leftmost frame) and λ = 1 represents the fully mutated sidechain (Lys-9, rightmost frame). For clarity, only six λ windows are shown, although significantly more are used in a typical FEP calculation.

**Figure 2:**
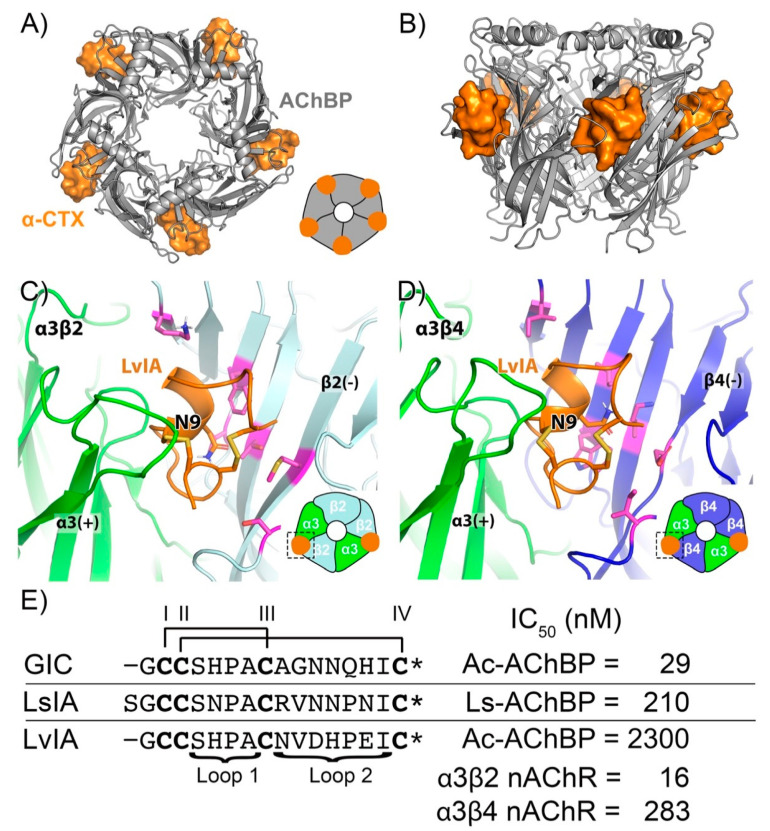
Overview of chemical systems: (**A**) extracellular view of LvIA (orange surface) and AChBP (gray cartoon); (**B**) transmembrane view of LvIA (orange surface) and AChBP (gray cartoon); (**C**) binding interface of LvIA and α3β2 nAChR; (**D**) binding interface of LvIA and α3β4 nAChR. LvIA (orange), AChBP (gray), α3 (green), β2 (pale cyan), and β4 (blue) are depicted in above images. Residues shown in pink differ between β subunits, lie within the binding interface of LvIA, and have a sidechain pointing towards the binding pocket (**E**) sequences of LvIA, LsIA, and GIC and their respective IC_50_s for different receptors. An asterisk (*) indicates an amidated C-terminus. Lines connecting cysteines labeled with Roman numerals indicate disulfide bonds.

Free-energy perturbation (FEP) is a rigorous computational method for estimating relative binding free energies (RBFE) that has been used to successfully predict the selectivity profiles of small molecules for kinases and phosphodiesterases [14,15]. For peptides, FEP can compute the RBFE between a wild-type and point mutant (ΔΔG_FEP_) via an “alchemical transformation” that “mutates” the wild-type (WT) sidechain to the mutant sidechain through a series of intermediates known as λ windows (Figure 1) [16,17]. Notably, FEP incorporates sampling of all degrees of freedom via molecular dynamics (MD) simulations to account for conformational variations in ligand–receptor interactions and permits the displacement and introduction of explicit waters during the simulation [18]. This contrasts with the widely used molecular mechanics–generalized born/surface area (MM-GB/SA) method, in which no alchemical transformation is performed, a static structure is used, and an implicit representation of the solvent is employed [19]. In principle, using FEP to predict the selectivity of conotoxin mutants at nAChRs is straightforward—FEP simulations of the conotoxin mutation of interest are run at the target and off-target nAChRs and then selectivity is calculated through the differences in the resulting ΔΔGs; however, to date no crystal or cryoelectron microscopy (Cryo-EM) structure of the pentameric nicotinic receptor ECD in complex with an α-conotoxin has been obtained [20]. As a result, low-resolution homology models of α-CTXs bound to nAChRs based on soluble homologues of the ECD of nAChRs [21,22], acetylcholine-binding protein (AChBP) from the mollusks *Aplysia californica* (Ac-AChBP) or *Lymnaea stagnalis* (Ls-AChBP), must be employed instead (Figure 2A,B). Ac-AChBP has low sequence identity to nAChR subtypes (<30%), but shares an overall architecture and key binding site residues with nAChRs and is highly amenable to co-crystallization with α-CTXs [23]. While such nAChR homology models can be successful at qualitatively rationalizing the selectivity profiles of α-CTX mutants [24,25], the ability to use them to quantitatively predict potency and selectivity is undetermined.

In this study, we use FEP to retrospectively predict potency and selectivity data for an archetypical system, the α-CTX LvIA from *Conus lividus*, which is naturally 18-fold more selective for the α3β2 nAChR than the highly similar α3β4 nAChR [26] (Figure 2C,D). The α3β2 nAChR is involved in a variety of physiological processes and the α3β4 nAChR is implicated in nicotine addiction [11]. This conotoxin and nAChR pair serves as a rigorous test for FEP selectivity calculations because the ECDs of the α3β2 and α3β4 nAChRs are 68% identical and point mutants of LvIA with a wide range of selectivity levels, some of which are counterintuitive, have been identified [26]. For example, LvIA[N9A] is >2000-fold more selective for the α3β2 nAChR than the α3β4 nAChR, although this mutant does not make significantly different contacts between the subtypes [26] (Figure 2C,D). We begin by examining the suitability of AChBP/α-CTX complexes as templates for nAChR/α-CTX homology models by using FEP and MM-GB/SA to retrospectively predict radioligand binding data for the conotoxins LvIA and GIC at Ac-AChBP and the conotoxin LsIA at Ls-AChBP [13,26,27] (Figure 2E). Second, we build homology models of the α3β2 and α3β4 nAChRs and retrospectively test the accuracy of FEP and MM-GB/SA in predicting the potency and selectivity of a set of point mutations of LvIA at these subtypes [26]. Third, in silico we mutate each non-cysteine position on LvIA to every genetically encoded amino acid (except cysteine) and use cloud-based FEP simulations to predict the selectivity levels of the resulting 225 mutants. Taken together, this study expands the domain of applicability of FEP to include selectivity calculations for α-CTXs and nAChRs, illustrates in principle how such an approach could be employed in a biologics drug discovery program devoted to this ion channel target and peptide modality, and identifies approaches for engineering selectivity into α-CTXs.

## 2. Results

### 2.1. Performance of FEP and MM-GB/SA on Mutagenesis Data

#### 2.1.1. Performance for AChBP

**Figure 3:**
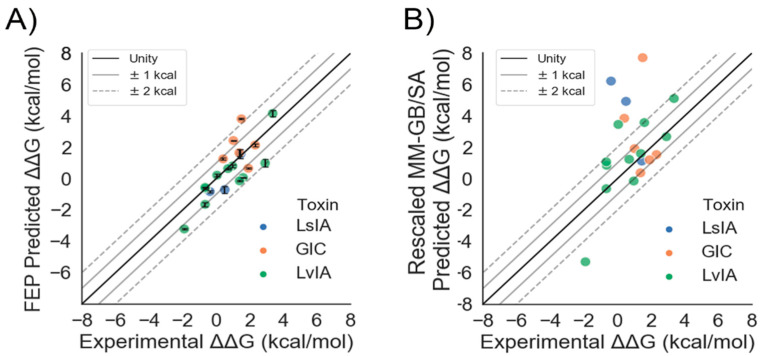
Quantitative prediction of the relative affinity of α-CTX mutants for AChBP: (**A**) scatter plot of ΔΔG_FEP_ vs. ΔΔG_EXP_; (**B**) scatter plot of ΔΔG_MM-GB/SA_ vs. ΔΔG_EXP_ with unity (solid, black line), ±1 kcal/mol error bands (solid gray lines), and ±2 kcal/mol error bands (dashed, gray lines) superimposed. The error bars show the standard error of the mean (SEM) from three independent FEP simulations.

First, we sought to examine the suitability of AChBP/α-CTX complexes as templates for nAChR/α-CTX homology models. Using a test set of twenty mutants for three different α-CTXs bound to AChBP receptors, we were able to re-predict the experimental affinities of the mutants relative to the WT (converted into ΔΔG_EXP_), with an overall root mean square error (RMSE) of 1.08 ± 0.15 kcal/mol and an R^2^ of 0.62 (Table 1 and Figure 3A) using FEP. MM-GB/SA was also used but performed worse, with an RMSE of 2.77 ± 0.54 kcal/mol and R^2^ of 0.18 (Table 1 and Figure 3B). The reported prime MM-GB/SA energies were rescaled by a factor of three in accordance with previous publications [19,28]. The performance by α-CTX is broken down in Table 2. LvIA bound to Ac-AChBP had comparable performance for FEP and MM-GB/SA, with RMSE values of 1.03 ± 0.19 kcal/mol and 1.83 ± 0.37 kcal/mol and R^2^ values of 0.76 and 0.60, respectively. GIC bound to Ac-AChBP had a lower RMSE value with FEP as compared to MM-GB/SA, with values of 1.27 ± 0.42 and 2.98 ± 1.03, respectively; however, for both methods, there were no correlations. For LsIA, MM-GB/SA predictions had an RMSE of 4.58 ± 1.62 kcal/mol and an R^2^ of 0.93, but an inverse correlation. For FEP, the RMSE was 0.75 ± 0.26 kcal/mol with an R^2^ of 0.80 when run using the OPLS3e force field. Interestingly, when the same mutations for LsIA were run with OPLS4, as performed for all other systems, the performance degraded, with a higher RMSE value of 2.34 ± 0.65 kcal/mol (Table S1).

#### 2.1.2. Performance for α3β2 and α3β4 nAChRs

**Figure 4:**
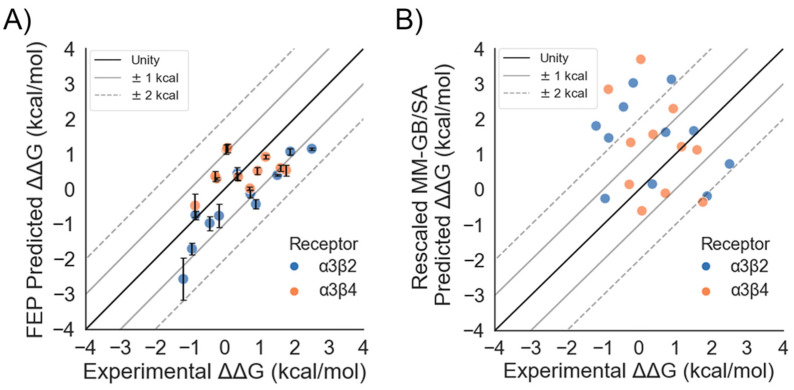
Quantitative prediction of the relative potency levels of LvIA mutants at the α3β2 and α3β4 nAChRs: (**A**) scatter plot of ΔΔG_FEP_ vs. ΔΔG_EXP_; (**B**) scatter plot of ΔΔG_MM-GB/SA_ vs. ΔΔG_EXP_ with unity (solid, black line), ±1 kcal/mol error bands (solid gray lines), and ±2 kcal/mol error bands (dashed, gray lines) superimposed. The error bars show the standard error of the mean (SEM) from three independent FEP simulations.

With the AChBP/α-CTX complexes validated by FEP, we proceeded to use these structures to build homology models of the α3β2 and α3β4 nAChRs in order to retrospectively test the ability of FEP to predict the potency and selectivity levels of various α-CTX point mutants bound to these nAChR subtypes. FEP was able to accurately predict the relative potencies of twenty-two mutations using homology models of the two subtypes, with an RMSE of 0.85 ± 0.08 kcal/mol and an R^2^ of 0.49 (Table 1 and Figure 4A). MM-GB/SA performed worse than FEP, with an RMSE of 1.96 ± 0.24 kcal/mol and an R^2^ of 0.06 (Table 1 and Figure 4B). For the α3β2 and α3β4 nAChRs, FEP re-predicted the experimental ΔΔGs, with RMSE values of 0.93 ± 0.11 kcal/mol and 0.77 ± 0.10 kcal/mol and R^2^ values of 0.82 and 0.12, respectively (Table 2). MM-GB/SA performed significantly worse, with an RMSE of 2.04 ± 0.26 kcal/mol and an R^2^ of 0.03 for the α3β2 nAChR and with an RMSE of 1.88 ± 0.47 kcal/mol and an R^2^ of 0.11 for the α3β4 nAChR.

#### 2.1.3. Performance of FEP by Type of Mutation

The performance of FEP and MM-GB/SA was also broken down by the type of mutation (Table 3). Charge-change mutations, which are typically difficult for FEP to predict [29], showed an RMSE of 0.82 ± 0.22 kcal/mol with FEP, performing better than MM-GB/SA that had an RMSE of 2.87 ± 0.67 kcal/mol. The correlation for charge-change mutations was higher than the overall R^2^ values for both FEP and MM-GB/SA, with R^2^ values of 0.79 and 0.22, respectively. Neutral mutations were also comparable to the overall FEP and MM-GB/SA performance, with RMSE values of 1.02 ± 0.10 kcal/mol and 2.11 ± 0.35 kcal/mol and an R^2^ values of 0.39 and 0.00, respectively. We also categorized the mutations by the differences between heavy atoms in the WT and mutant residue. When mutating a bigger residue to a smaller one, FEP and MM-GB/SA had RMSE values of 0.95 ± 0.09 kcal/mol and 2.06 ± 0.22 kcal/mol, respectively. Small residues that were mutated to bigger residues had a higher RMSE for FEP, with a value of 1.41 ± 0.40 kcal/mol. The RMSE for MM-GB/SA was also higher, with a value of 3.00 ± 1.04 kcal/mol. Mutations that had no difference in heavy atoms had an RMSE of 0.87 ± 0.14 kcal/mol for FEP but a value of 3.36 ± 1.16 kcal/mol for MM-GB/SA.

**Figure 5:**
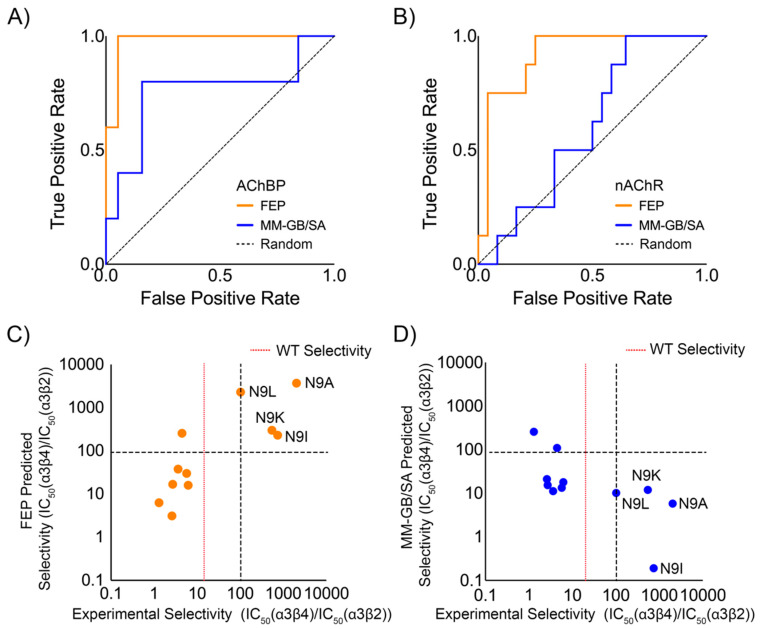
Classification of potency- and selectivity-enhancing LvIA mutants: (**A**) ROC plot comparing the ability of FEP and MM-GB/SA to classify mutations to LvIA, GIC, and LsIA that gain affinity for AChBP relative to WT; (**B**) ROC plot comparing the ability of FEP and MM-GB/SA to classify LvIA mutations that gain potency for the α3β2 or α3β4 nAChR relative to WT; (**C**) classification of the selectivity of LvIA mutants by FEP; (**D**) classification of the selectivity of LvIA mutants by MM-GB/SA.

FEP also performed better than MM-GB/SA in classifying mutations as having a gain in potency or affinity (ΔΔG < 0 kcal/mol) versus a loss in potency or affinity (ΔΔG > 0 kcal/mol). The area under the curve (AUC) of a receiver operating characteristic (ROC) plot for FEP was 0.94, with a statistically significant *p*-value of <0.01 and a 95% confidence interval (CI) of 0.88 to 1.0 (Table 4), whereas MM-GB/SA performed worse, with an AUC of 0.66 with a p-value of 0.09 (Table 4). FEP had an accuracy of 88%, while MM-GB/SA had an accuracy of 71%. For twenty-four mutations at AChBP, FEP had an AUC of 0.98, as compared to MM-GB/SA with an AUC of 0.76 (Figure 5A). For mutations at nAChRs, FEP performed significantly better than MM-GB/SA, with AUC values of 0.92 and 0.60, respectively (Figure 5B).

### 2.2. Performance in Classifying Selective LvIA Mutants

We next assessed the ability of FEP and MM-GB/SA to accurately classify LvIA mutants >100X selective for the α3β2 nAChR over the α3β4 nAChR. FEP was able to correctly classify all four selective mutants, namely LvIA[N9L], LvIA[N9K], LvIA[N9I], and LvIA[N9A] (Figure 5C), with only one mutation, Lv1A[V10A], being incorrectly classified as selective. In contrast, MM-GB/SA predicted all four selective mutants as unselective (Figure 5D). The most notable mutation is LvIA[N9A], which has an experimentally measured 2032-fold selectivity [26], with experimental ΔΔG values of −1.19 kcal/mol and 1.61 kcal/mol at the α3β2 and α3β4 nAChRs, respectively. FEP correctly predicted this selectivity, with a predicted ΔΔG of −2.56 ± 0.61 kcal/mol at the α3β2 nAChR and 0.60 ± 0.08 kcal/mol at the α3β4 nAChR, equivalent to a predicted 3690-fold selectivity. In contrast, MM-GB/SA predicted LvIA[N9A] to have a ΔΔG of 1.81 kcal/mol at the α3β2 nAChR and ΔΔG of 1.13 kcal/mol at the α3β4 nAChR, equivalent to a predicted 6-fold selectivity, which is an underestimate by about three orders of magnitude. Overall, FEP had an accuracy of 91% in classifying mutations as being selective or non-selective, with a significant *p*-value of 0.015, as calculated by Fisher’s exact test, whereas MM-GB/SA had an accuracy of 55%, which was not statistically significant. We also tested how sensitive the MM-GB/SA accuracy was to the specific protein conformation employed by repeating the calculations with poses of the α3β2 and α3β4 nAChRs extracted from three different points along the homology modeling and simulation workflows (Figure S1). In all cases, the accuracy was less than 65%, and at most half of the selective mutations were correctly identified. Performing the MM/GB-SA calculations using an ensemble of ten poses did lead to the correct classification of LvIA[N9K] as selective, but overall did not result in a statistically significant improvement in accuracy (Figure S2).

**Figure 6:**
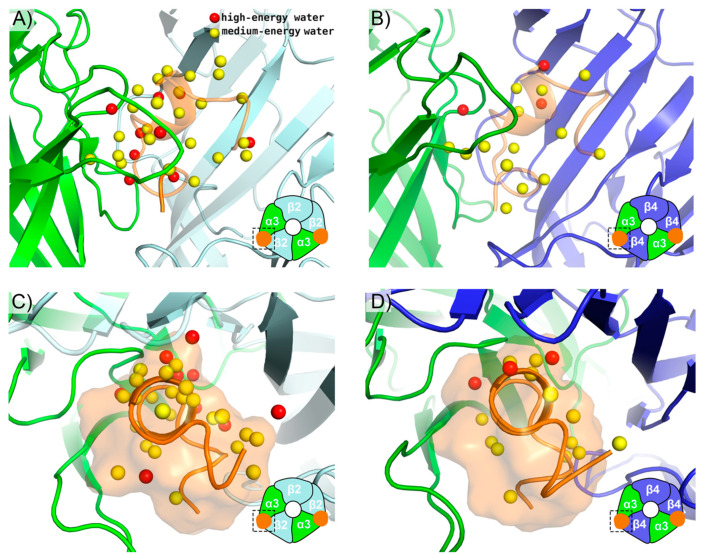
WaterMaps of nAChR subtypes. The apo (**A**) α3β2 nAChR binding site and (**B**) α3β4 nAChR binding site are shown with their respective WaterMaps. LvIA is shown as an orange, semi-transparent cartoon for reference but is not present during the WaterMap simulations. (**C**) Extracellular view of α3β2 nAChR binding site and WaterMap (**D**) Extracellular view of α3β4 nAChR binding site and WaterMap. A semi-transparent orange surface is shown around LvIA. Medium-energy water sites with predicted ΔG > 1.5 kcal/mol are colored yellow and high-energy water sites with predicted ΔG > 3.5 kcal/mol are colored red. For clarity, only medium-energy or high-energy water sites that overlap with the position of LvIA in the bound state are shown.

Although FEP can correctly classify selective mutations, understanding the structural basis for the selectivity of LvIA for the α3β2 nAChR over the α3β4 nAChR could provide insight for future mutagenesis studies. Because the 18-fold selectivity of LvIA for the α3β2 nAChR cannot be readily explained by the differential interactions it makes between the two subtypes (Figure 2C,D), we hypothesized that computing and visualizing the water thermodynamic maps in the binding sites of the two subtypes could explain the selectivity of LvIA. To test this hypothesis, “apo” WaterMap simulations (conotoxin not present) were run for both the α3β2 nAChR and α3β4 nAChR and the locations of the unstable water sites (medium-energy or high-energy) were compared to the pose of LvIA. WaterMap placed a total of 61 water sites within 3 Å of LvIA at α3β2 nAChR and a total of 66 water sites within 3 Å of LvIA at the α3β4 nAChR, allowing us to compare the two WaterMaps further and investigate differences in the water site energetics. At the α3β2 nAChR, thirty-three of these unstable water sites overlapped with the binding mode of LvIA (overlap factor greater than 0.1) (Figure 6A,C) versus seventeen at the α3β4 nAChR (Figure 6B,D). Taken together, these results suggest that LvIA displaces more and higher-energy unstable waters when binding to the α3β2 nAChR than the α3β4 nAChR, which could account for why it is more potent in the former subtype. These findings are consistent with a previous study in which water thermodynamics was used to explain mutagenesis data for a variety of peptide toxins for different ion channels [12].

### 2.3. In Silico Scan for Putative Selectivity-Enhancing Mutations with FEP

**Figure 7:**
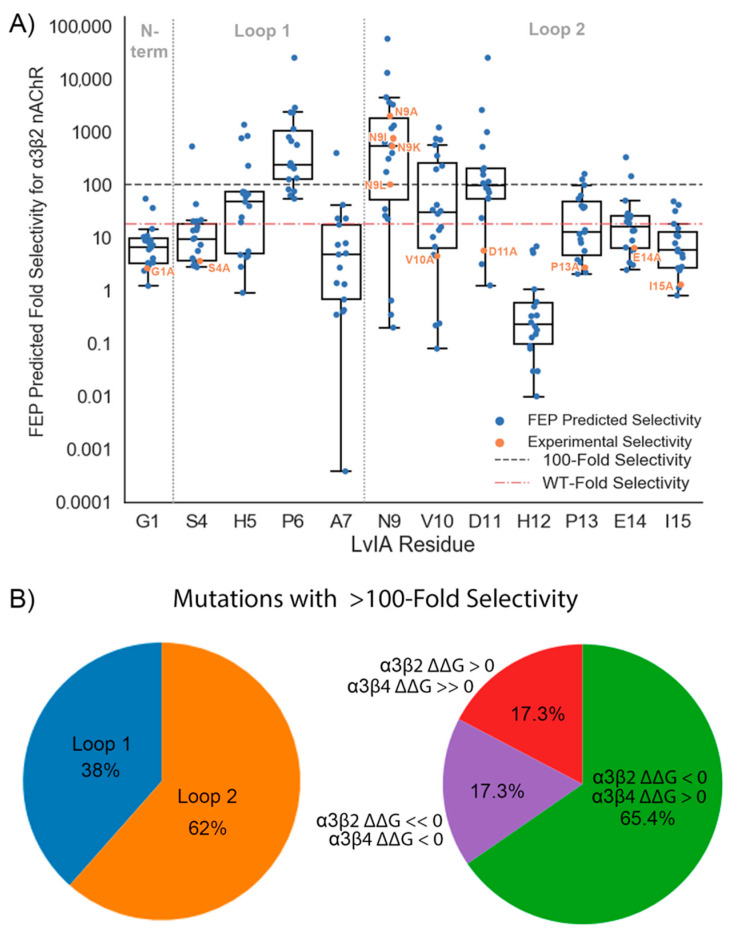
In silico exhaustive mutagenesis of LvIA: (**A**) Fold selectivity levels of LvIA point mutants predicted by FEP (blue points) and measured experimentally (orange points) are plotted by residue, with box plots overlaid. The black dashed line denotes the cutoff for a mutation to be considered selective (100X), while the red dot-dashed line denotes the fold selectivity of the WT LvIA (18X); (**B**) Pie charts show the compositions of point mutations predicted to be selective by the loop they are on (left panel) and by their predicted ΔΔGs at each nAChR subtype (right panel).

Although an alanine scan of LvIA succeeded in identifying a mutation such as LvIA[N9A] that is ~2000X selective [26], we were curious to see if mutations with an even greater degree of selectivity could be identified in silico. To address this question, an exhaustive amino acid scan of LvIA was performed. Each non-cysteine position on LvIA was mutated to every amino acid (except cysteine). We then predicted the ΔΔG_FEP_ values of these 225 point mutations at both the α3β2 and α3β4 nAChRs and the resulting selectivity ratios (Figure 7). Our scan predicted selective mutations at nine different residues, including all four residues in loop 1 and five in loop 2. No mutations were predicted to be selective at the N-terminal LvIA[G1]. In loop 1, 6% of mutations at LvIA[S4], 21% of mutations at LvIA[H5], 78% of mutations at LvIA[P6], and 6% of mutations at LvIA[A7] were predicted to be selective. In loop 2, 67% of mutations at LvIA[N9], 39% of mutations at LvIA[V10], 53% of mutations at LvIA[D11], no mutations at LvIA[H12], 11% of mutations at both LvIA[P13] and LvIA[E14], and no mutations at LvIA[I15] were predicted to be selective (Figure 7A). Overall, out of 225 mutations, fifty-two were predicted to be >100X selective, with four predicted to be >10,000X selective. Of the mutations predicted to be selective, 38% were located on loop 1 and 62% were located on loop 2 (Figure 7B, left panel). We also examined the ΔΔG_FEP_ at the two receptors to understand why these mutations were predicted to be selective (Figure 7B, right panel). Overall, 65% were predicted to be selective due to an increase in potency at the α3β2 nAChR (ΔΔG_FEP_ < 0) and decrease in potency at the α3β4 nAChR (ΔΔG_FEP_ > 0). Interestingly, nine mutations predicted to be selective had a gain of potency at both receptors, but the magnitude of the gain was much larger at the α3β2 nAChR than at the α3β4 nAChR (ΔΔG_FEP_(α3β2) << 0, ΔΔG_FEP_(α3β4) < 0). For example, a charge-change mutation at position ten on LvIA had a predicted ΔΔG of −2.12 kcal/mol at the α3β2 nAChR and a predicted ΔΔG of −0.84 kcal/mol at the α3β4 nAChR. Finally, nine mutations were predicted to be selective due to a loss in potency at both receptors, but with a much greater loss in potency at the α3β4 nAChR than at the α3β2 nAChR (ΔΔG_FEP_(α3β2) > 0, ΔΔG_FEP_(α3β4) >> 0) (Figure 7B, right panel). For example, a mutation at position five on LvIA had predicted ΔΔG values of 1.02 kcal/mol and 3.3 kcal/mol at the α3β2 and α3β4 nAChRs, respectively. Although these findings remain to be experimentally validated, taken together they suggest that additional highly selective mutations for LvIA may exist.

## 3. Discussion

The ability to accurately predict how a mutation to an α-CTX will affect its potency and selectivity for nAChRs is a “grand challenge” in the field [30]. Computational methods have the potential to help meet this challenge, but their ability to recapitulate known data must be rigorously assessed using challenging test cases before they can be used prospectively [12,31]. Here, we performed such a study by examining the ability of FEP to retrospectively predict a wide range of potency and selectivity levels of LvIA mutants for the highly similar α3β2 and α3β4 nAChRs.

### 3.1. FEP Quantitatively Predicts the Relative Changes in Free Energy of Conotoxin Mutants for AChBPs and nAChRs with Accuracy

We sought to build on the previous success in finding a correlation between measured and predicted potency levels [32,33] of α-CTX mutants for nAChRs by quantitatively predicting the magnitudes and signs of the ΔΔGs due to the mutations. For the two nAChRs, FEP gave an overall RMSE of 0.85 ± 0.08 kcal/mol and an R^2^ of 0.49, suggesting this aim was achieved (Table 1 and Figure 4). In contrast, the more widely used MM-GB/SA method gave an RMSE of 1.96 ± 0.24 kcal/mol and R^2^ of 0.06 (Table 1 and Figure 4). These results are consistent with the emerging view that accounting for the dynamics of α-CTX/nAChR interactions, as is the case in FEP, is necessary to accurately model them [30,34,35]. They are also consistent with the results of a similar study in which FEP was retrospectively applied to homology models of small molecules bound to proteins [36].

Of the five systems modeled in this study, LsIA/Ls-AChBP required additional effort to be modeled accurately. The LsIA/Ls-AChBP system was purposefully included, despite only having three mutational data points, because it is the sole example of an α-CTX crystallized in complex with Ls-AChBP. From a structural biology perspective, the LsIA[R10F] and LsIA[R10M] mutations involve altering a complex network of contacts between WT LsIA[R10] and Ls-AChBP that include a cation-π interaction with Ls-AChBP[Y164] and a salt bridge interaction with Ls-AChBP[D160], the latter of which may be influenced by crystal contacts [27]. FEP predictions on these mutations were degraded with the OPLS4 force field [37] compared to the OPL3e forcefield [38] (Table S1). This was likely caused by a reduction in the salt bridge strength in the updated parameterization and the absence of an explicit cation-π term in the forcefield [39].

Finally, two specific sets of mutations also proved difficult for FEP. The first was the GIC/Ac-AChBP system, for which the R^2^ value was 0 and the RMSE was 1.27 ± 0.42 kcal/mol. Although this RMSE is within the error range of the 1 kcal/mol RMSE considered desirable for FEP models, the R^2^ value may be low due to the small sample size and dynamic range of the data [40]. FEP also performed less well for the ‘small-to-big’ group of mutations, with an R^2^ value of 0.24 and an RMSE of 1.41 ± 0.40 kcal/mol. This could reflect the fact that mutations that gain size can lead to protein reorganization, which is difficult to sample on the timescale of FEP simulations [17]. Nonetheless, these caveats should not obscure the main finding of this study, which is that overall FEP can accurately retrospectively predict the free-energy changes of α-CTX mutants for nAChRs.

### 3.2. FEP Accurately Classifies Conotoxin Mutations That Enhance Selectivity for an nAChR

Overall, FEP was able to correctly classify conotoxin mutations that gain selectivity for one nAChR subtype over another. We found that FEP classified all four LvIA mutants with >100X fold selectivity for the α3β2 nAChR over the α3β4 nAChR as true positives at the cost of only one false positive prediction, with an overall accuracy of 91% (Figure 5). In contrast, MM-GB/SA did not identify any selective mutations correctly and had an overall accuracy of 55%. The ability of FEP to classify selectivity-enhancing mutations is an indication of the method’s predictive power and suggests it might be complementary to experimental methods, such as alanine scanning, which are expensive and time-consuming. Future studies may focus on going beyond the classification of selectivity to quantitatively predicting its magnitude. However, this is an intrinsically more difficult problem due to the propagation of uncertainty in the final selectivity prediction (i.e., the difference between two predictions with 1 kcal/mol error each will have a propagated error of 1.4 kcal/mol if they are uncorrelated) [14]. Higher-resolution α-CTX structures complexed with different nAChR ECD subtypes may enable such calculations. More broadly, our dynamics-based approach in FEP for computing selectivity is in line with a similar study that found that inclusion of multiple frames in MM-GB/SA calculations could be an important factor in prospectively identifying selective mutations of the conotoxin RegIIA for the α3β2 nAChR over the α3β4 nAChR [41].

### 3.3. An Exhaustive In Silico Scan Predicts Additional Selectivity-Enhancing Point Mutations May Exist for LvIA

We assessed our ability to computationally identify putative selectivity-enhancing mutations by exhaustively mutating LvIA at each position to every (permissible) genetically encoded amino acid, except cysteine, and used FEP to predict the resulting ΔΔGs at both nAChR subtypes. Out of 225 mutations, 23% were predicted to have >100X selectivity for the α3β2 nAChR over the α3β4 nAChR (Figure 7A). In general, our results are in accordance with previous studies and findings. For example, 67% of prospective mutations to LvIA[N9] were predicted >100X selective (Figure 7A), in agreement with the critical role that this residue is known to play in enhancing selectivity for LvIA and other 4/7 α-CTXs [26]. Furthermore, consistent with the hypothesis that residues on loop 2 govern subtype selectivity [42], 62% of the mutations predicted to be selective were present on loop 2 (Figure 7B). One unexpected finding that emerged was that 78% of mutations made to LvIA[P6], which is located on loop 1, were predicted to have some degree of selectivity (Figure 7A). Since proline at this position is highly conserved amongst α-CTXs [10], these predictions are counterintuitive and could be false positives; however, given the excellent retrospective performance of FEP in classifying selective mutations and the fact that proline mutants were not simply indiscriminately predicted to be selective (e.g., those at LvIA[P13] were not), these mutations may warrant future experimental investigation. 

Finally, our large-scale in silico scan revealed new strategies for engineering selective α-CTXs. While we found that the majority of the mutations predicted to be selective had the “expected” changes in potencies at the two subtypes (predicted gain in potency for α3β2 nAChR and loss in potency for α3β4 nAChR), two less conventional possibilities emerged as well. In 17% of the cases, predicted selectivity was achieved through loss of potency at both subtypes, although the magnitude of the loss was predicted to be much greater at the α3β4 nAChR. In contrast, a predicted gain in potency for both subtypes, with a larger gain for α3β2 nAChR, was also observed 17% of the time. Taken together, these findings suggest alternate ways to engineer selectivity into conotoxins beyond mutating residues to attempt to “clash” with the off-target nAChR subtype. More generally, computational methods that embrace the dynamics of α-CTX/nAChR interactions [30,41] are increasingly being used to prospectively identify selectivity-enhancing mutations. With the passage of α-CTX antagonists of nAChRs towards clinical trials [43,44,45], our findings set the stage for the prospective use of FEP to advance such drug discovery efforts.

## 4. Materials and Methods

### 4.1. AChBP Protein Preparation

All calculations were performed using the 2021-1 release of Maestro (Schrödinger, Inc., New York, NY, USA), unless otherwise noted. LsIA and Ls-AChBP (PDB: 5T90), LvIA and Ac-AChBP (PDB: 5XGL), and GIC in complex with Ac-AChBP (PDB: 5CO5) were all downloaded from the Protein Data Bank (PDB). For the LsIA/Ls-AChBP structure (PDB: 5T90), the model was manually inspected to adjust sidechain rotamers and rebuild any poorly resolved loops with Coot [46], followed by a round of macromolecular structure refinement with Phenix/OPLS3e (a version of Phenix [47], whereby the OPLS3e force field [38] and VSGB2.1 solvation model [48] are used to calculate energies and gradients; 2020-3 release of Maestro). Each structure was aligned and truncated to include two receptor chains and one toxin bound. The Protein Preparation Wizard was used to cap the N- and C-termini with acetyl and N-methyl amide groups, respectively. Missing sidechains and loops were filled in using Prime. Protonation states were assigned using PROPKA at pH 7.4 and hydrogen bond networks were optimized using the “H-bond assignment” panel. Restrained minimization was carried out using the OPLS4 force field [33], with heavy atoms converged to a root mean square deviation (RMSD) of 0.3 Å.

### 4.2. nAChR Homology Model Construction

Homology models were built using the ‘build homology model’ panel in the Multiple Sequence Viewer/Editor Panel in Maestro. The target sequence was imported from Uniprot using the respective ECD sequence for *Rattus norvegicus* β2 (P12390), *Rattus norvegicus* β4 (P12392), or *Rattus norvegicus* α3 (P04757). The template structure used was 5XGL after preparation, as described in the previous section. *Rattus norvegicus* β2 and β4 sequences were each aligned to 5XGL chain A, while the *Rattus norvegicus* α3 sequence was aligned to 5XGL chain B. The LvIA peptide sequence (L8BU87) was used as the target sequence and aligned to 5XGL chain C.

The initial homology model for each subtype was then subject to refinement. Using the ‘protein–protein’ selection tool, all residues at the binding interface between the conotoxin, principal subunit, and complementary subunit were selected and refined using the ‘predict sidechains’ panel in Maestro. Once sidechain prediction was completed, the structure underwent the protein preparation protocol described in Section 4.1, except only hydrogens were subjected to restrained minimization. Next, an MD simulation was performed to ensure structural integrity and resolve any remaining steric clashes. Using the ‘system builder’ panel, an SPC solvent model was placed on the structure. No neutralizing counterions or salt were added. An MD simulation with Desmond (Desmond Molecular Dynamics System, D. E. Shaw Research, New York, NY, USA, 2020. Maestro-Desmond Interoperability Tools, Schrödinger, New York, NY, USA, 2020) was performed for 15 ns on 4 GPUs on a GPU cluster consisting of NVIDIA Pascal-generation GPUs. Following manual inspection, a single representative frame without steric clashes and with low RMSD to the starting model was then selected from the trajectory (the 19th frame for the α3β2 nAChR and the 1st frame for the α3β4 nAChR). These frames were then used as inputs for FEP and MM-GB/SA calculations.

### 4.3. Selection of Mutants

Forty-two IC_50_’s due to mutations to LsIA, GIC, and LvIA were gathered from three sources [13,26,27] and all mutations with reported IC_50_ values were used for FEP benchmarking. Fourteen additional IC_50_’s due to mutations were used to assess classifier performance but not RMSEs, because they were qualified data points at the top of the assay. To convert reported IC_50_ values to ΔΔG_EXP,_ the relation ΔΔG_EXP_ = R × T × ln(IC_50_(MUT)/IC_50_(WT)) was used, in which IC_50_(MUT) is the IC_50_ of the mutant peptide, IC_50_(WT) is the IC_50_ of the WT (unmutated) peptide, R is the universal gas constant, and T is the temperature at 298 K with R × T = 0.593 kcal/mol. The specific WT IC_50_ measured in each study was used when converting that study’s mutational data into free energies.

### 4.4. WaterMap Calculations

WaterMap calculations were set up and run using the WaterMap panel in Maestro as previously described [12]. The toxin chain was selected as the ligand and waters within 10 Å of the selected ligand were analyzed. An “apo” (toxin not retained in calculations) WaterMap was run. For the WaterMap analysis, the overlap factor was set to 0.1 to identify water sites that overlap with the coordinates of LvIA. A custom script was then used to categorize water sites based on their free energy. Medium-energy water sites were colored yellow (1.5 < ΔG < 3.5 kcal/mol) and high-energy water sites were colored red (ΔG ≥ 3.5 kcal/mol) [12].

### 4.5. RBFE Calculations with MM-GB/SA

MM-GB/SA calculations were set up in the ‘residue scanning’ panel. After undergoing the refinement procedure described in Section 4.2, the structure was imported into the ‘residue scanning’ panel and the ‘stability and affinity’ calculation type was selected. The toxin chain was chosen to bind to the other two chains in the input structure. Default settings were used for all residue scanning calculations, along with a 0 Å cutoff for sidechain prediction with backbone minimization (only the residue being mutated was permitted to repack). The predicted affinities calculated by MM-GB/SA were then rescaled as described previously by dividing each predicted ΔΔG by a factor of three [19,28]. Additionally, MM-GB/SA calculations were repeated using an ensemble of ten evenly spaced frames selected from the 25 ns MD trajectory of the WT LvIA FEP simulation (described in Section 4.6). The RBFE was then calculated over this ensemble using an in-house script, thermal_mmgbsa.py [49].

### 4.6. RBFE Calculations with FEP

Retrospective FEP calculations were carried out as follows. The same input structure used for MM-GB/SA calculations was imported into the Protein FEP panel in Maestro and the selectivity calculation type was selected. For each system, the toxin chain was chosen to bind to the two receptor chains. Defaults were used for all parameters other than the simulation time, which was increased to 25 ns, as well as the number of λ windows, which was set to 24 for all perturbation types. The FEP job was run on 4 GPUs on a GPU cluster consisting of NVIDIA Pascal-generation GPUs. Finally, when performing charge-change mutations with FEP, it can be important to perturb to the neutral form of the residue when it is in close proximity to other charged residues and a hydrogen bond network is involved [28]; therefore, for the LsIA[N9D] mutation, N9 was mutated into protonated Asp (ASH), while for the LvIA[N9K] mutation at both the α3β2 nAChR and α3β4 nAChR, N9 was mutated into neutral Lys (LYN). Finally, FEP calculations for LsIA/Ls-AChBP were also performed as described above but using the 2020-3 release of Maestro, which employs the OPLS3e forcefield [38].

### 4.7. Point Mutation Scan

An amino acid scan was performed at LvIA, in which every non-cysteine residue was mutated into every residue except cysteine, and the resulting RBFEs at the α3β2 nAChR and α3β4 nAChR were computed with FEP. The structure file was imported into the FEP panel and the selectivity calculation was selected. The toxin chain was chosen to bind to the two receptor chains. For each residue, all standard amino acid mutations that were possible for that residue were selected. This resulted in a total of 225 mutations for LvIA at both the α3β2 and α3β4 nAChRs. Mutations to histidine were performed for all three tatuomer and charge forms (HID, HIE, HIP) and the state predicted as being most selective upon mutation was used for plotting and analysis. Default parameters were used with the exception of simulation time, which was set to 20 ns, as well as the number of λ windows, which was set to 24 for all perturbation types. The FEP job ran on FEP+ Web Services, which is a service provided by Schrödinger to run FEP calculations on cloud computing resources using NVIDIA Tesla-generation GPUs.

### 4.8. Selectivity Calculations

Upon completion of FEP and MM-GB/SA RBFE calculations at the α3β2 nAChR and α3β4 nAChR subtypes, the predicted α3β4/α3β2 selectivity ratio, R, was computed using the relation R = F × exp(ΔSelectivity/0.593), where F = IC_50_(α3β4)/IC_50_(α3β2) for the WT LvIA and ΔSelectivity = ΔΔG_MUT_(α3β4) − ΔΔG_MUT_(α3β2) in kcal/mol.

### 4.9. Statistics

Statistical analysis of the FEP results and their comparison to experimental data were performed following accepted best practices [50]. To minimize the effects of trial-to-trial variability in ΔΔG_FEP_, every retrospective mutation was run in triplicate with a different random seed and the ΔΔG_FEP_s from each of the three independent simulations were averaged to arrive at a mean ΔΔG_FEP_, which was used in all analyses. Bootstrapped estimates for RMSE and MUE were calculated using the FEP+ panel software in Maestro. For binary classification, the AUC of a ROC plot and its associated CI were computed using Prism 9 with default options (GraphPad Software: San Diego, CA, USA). Mutations with a ΔΔG_EXP_ < 0 were classified as having a gain in potency. Mutations with IC_50_(α3β4)/IC_50_(α3β2) > 100 were classified as selective.

## Figures and Tables

**Table 1:** Performance by receptor.

Receptor	Number of Mutations	Potency Range (kcal/mol)	FEP		MM-GB/SA	
			R^2^	RMSE	R^2^	RMSE
AChBP	20	−1.90–3.66	0.62	1.08 ± 0.15	0.18	2.77 ± 0.54
nAChR	22	−1.19–2.51	0.49	0.85 ± 0.08	0.06 *	1.96 ± 0.24
Total	42	−1.90–3.66	0.58	0.96 ± 0.09	0.07	2.37 ± 0.31

* Negative correlation coefficient.

**Table 2:** Performance by conotoxin.

Toxin	Receptor	Number of Mutations	Potency Range (kcal/mol)	FEP		MM-GB/SA	
				R^2^	RMSE	R^2^	RMSE
	Ac-AChBP	11	−1.90–3.36	0.76	1.03 ± 0.19	0.60	1.83 ± 0.37
LvIA	α3β2 nAChR	11	−1.19–2.51	0.82	0.93 ± 0.11	0.03 *	2.04 ± 0.26
	α3β4 nAChR	11	−0.85–1.77	0.12	0.77 ± 0.10	0.11 *	1.88 ± 0.47
GIC	Ac-AChBP	6	0.41–2.32	0.00	1.27 ± 0.42	0.05 *	2.98 ± 1.03
LsIA	Ls-AChBP	3	−0.38–1.45	0.80	0.75 ± 0.26	0.93 *	4.58 ± 1.62

* Negative correlation coefficient.

**Table 3:** Performance by charge and size change.

Type of Mutation	Number of Mutations	Potency Range (kcal/mol)	FEP		MM-GB/SA	
			R^2^	RMSE	R^2^	RMSE
By charge						
Charge-Change	13	−1.90–3.36	0.79	0.82 ± 0.22	0.22	2.87 ± 0.67
Neutral	29	−1.19–2.51	0.39	1.02 ± 0.10	0.00	2.11 ± 0.35
By size						
Big-to-Small	32	−1.90–3.36	0.60	0.95 ± 0.09	0.16	2.06 ± 0.22
Small-to-Big	5	0.73–1.89	0.24	1.41 ± 0.40	0.07	3.00 ± 1.04
No change in heavy atoms	5	−0.94–1.77	0.48	0.87 ± 0.14	0.11 *	3.36 ± 1.16

* Negative correlation coefficient.

**Table 4:** Performance in classifying gain of potency mutations.

Receptor	Number of Mutations	Potency Range (kcal/mol)	FEP AUC	MM-GB/SA AUC
AChBP	24	−1.90–3.66	0.98 (0.93 to 1.0)	0.76 (0.47 to 1.0)
nAChR	32	−1.19–3.83	0.92 (0.82 to 1.0)	0.60 (0.40 to 0.80)
Total	56	−1.90–3.83	0.94 (0.88 to 1.0)	0.66 (0.49 to 0.82)

## Supplementary Materials

The following are available online at https://www.mdpi.com/article/10.3390/md19070367/s1, Figure S1: MM-GB/SA selectivity predictions for LvIA mutants using different nAChR conformations, Figure S2: Performance of MM-GB/SA using an ensemble of conformations, Table S1: Comparison of FEP affinity predictions for LsIA mutations using OPLS3e and OPLS4 forcefields.
