# Supplementary material for: Potency- and Selectivity-Enhancing Mutations of Conotoxins for Nicotinic Acetylcholine Receptors Can Be Predicted Using Accurate Free-Energy Calculations
Source: Mar Drugs. 2021 Jun 25;19(7):367. doi: 10.3390/md19070367 (PMC8306581; doi:10.3390/md19070367)
Supplement: Supplementary file 1 [file marinedrugs-19-00367-s001.zip › marinedrugs-1256164-SI updated.pdf]

marine drugs

Article

Potency- and Selectivity-Enhancing Mutations of Conotoxins for Nicotinic Acetylcholine Receptors can be Predicted Using Accurate Free-Energy Calculations

Dana Katz[1], Michael A. DiMattia[1], Dan Sindhikara[1,†], Hubert Li[1], Nikita Abraham[2], and Abba E. Leffler[1*]

[1] Schrödinger, Inc., 120 West 45th St., New York, NY 10036, United States
[2] D.E. Shaw India Private LtD., Hyderabad, Telangana 500096, India
* Correspondence: abba.leffler@schrodinger.com
† Current address: Merck and Co., Inc., Kenilworth, NJ 07033, United States

Table of Contents

Table S1. Comparison of FEP affinity predictions for LsIA mutations using OPLS3e and OPLS4 forcefields
Figure S1. MM-GB/SA selectivity predictions for LvIA mutants using different nAChR conformations
Figure S2. Performance of MM-GB/SA using an ensemble of conformations

Table S1. Comparison of FEP affinity predictions for LsIA mutations using OPLS3e and OPLS4 forcefields

LsIA Mutation	Experimental ΔΔG at Ls-AchBP (kcal/mol)	OPLS3e Predicted ΔΔG (kcal/mol)	OPLS4 Predicted ΔΔG (kcal/mol)
R10M	0.51	-0.70 ± 0.22	-2.72 ± 0.18
R10F	-0.38	-0.82 ± 0.07	-2.81 ± 0.34
N12Q	1.45	1.57 ± 0.30	1.25 ± 0.11

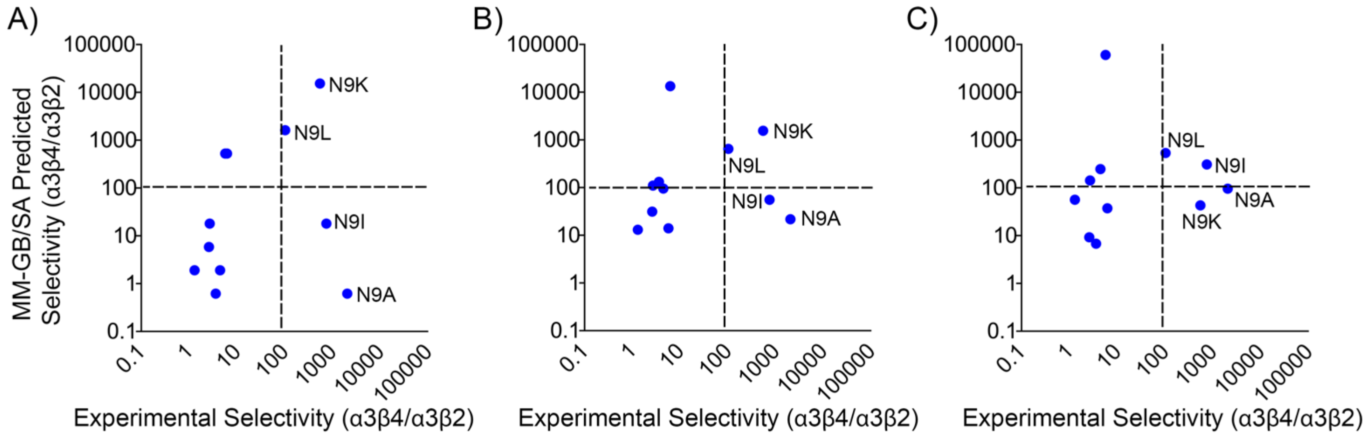

Figure S1. MM-GB/SA selectivity predictions for LvIA mutants using different nAChR conformations. The accuracy of MM/GB-SA at classifying LvIA mutants as selective or not was measured using α3β2 and α3β4 nAChR conformations **(A)** prior to MD refinement **(B)** extracted from the respective simulation frames after 5 ns of MD refinement **(C)** extracted from the final frames of the respective WT LvIA FEP trajectories. The four mutants which are experimentally verified to be greater than 100X selective are labeled.

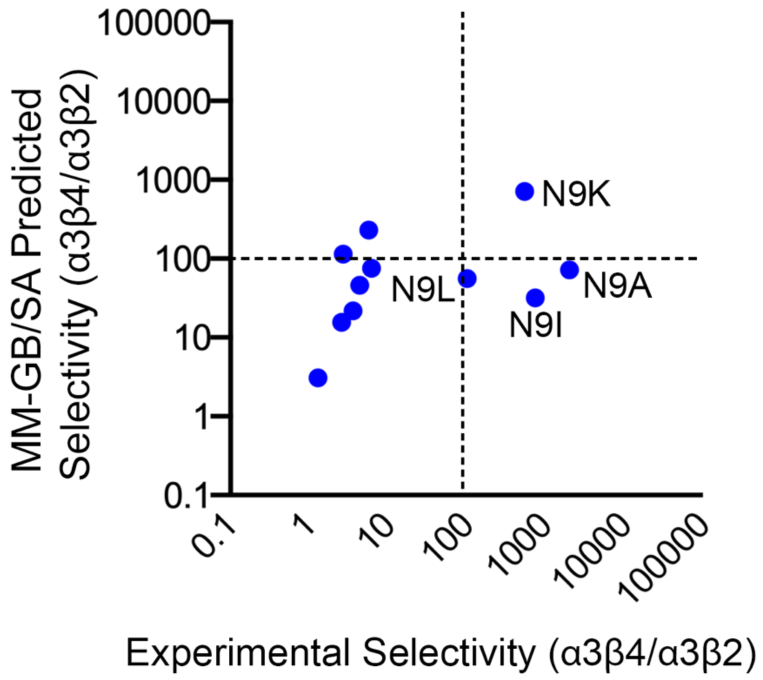

Figure S2. Performance of MM/GB-SA using an ensemble of conformations. The selecitives of eleven LvIA mutants were computed with MM/GB-SA using ten, evenly spaced frames from a 25 ns MD trajectory. The four mutants which are experimentally verified to be greater than 100X selective are labeled.
